# Full-Color Biomimetic Photonic Materials with Iridescent and Non-Iridescent Structural Colors

**DOI:** 10.1038/srep33984

**Published:** 2016-09-23

**Authors:** Ayaka Kawamura, Michinari Kohri, Gen Morimoto, Yuri Nannichi, Tatsuo Taniguchi, Keiki Kishikawa

**Affiliations:** 1 Division of Applied Chemistry and Biotechnology, Graduate School of Engineering, Chiba University, 1-33 Yayoi-cho, Inage-ku, Chiba 263-8522, Japan; 2 Yamashina Institute for Ornithology, 115 Konoyama, Abiko, Chiba 270-1145, Japan

## Abstract

The beautiful structural colors in bird feathers are some of the brightest colors in nature, and some of these colors are created by arrays of melanin granules that act as both structural colors and scattering absorbers. Inspired by the color of bird feathers, high-visibility structural colors have been created by altering four variables: size, blackness, refractive index, and arrangement of the nano-elements. To control these four variables, we developed a facile method for the preparation of biomimetic core-shell particles with melanin-like polydopamine (PDA) shell layers. The size of the core-shell particles was controlled by adjusting the core polystyrene (PSt) particles’ diameter and the PDA shell thicknesses. The blackness and refractive index of the colloidal particles could be adjusted by controlling the thickness of the PDA shell. The arrangement of the particles was controlled by adjusting the surface roughness of the core-shell particles. This method enabled the production of both iridescent and non-iridescent structural colors from only one component. This simple and novel process of using core-shell particles containing PDA shell layers can be used in basic research on structural colors in nature and their practical applications.

Coloration is one of the most interesting aspects in nature^1^,^2^. The beautiful structural colors in bird feathers are produced by pigments, such as carotenoids (pigment color), and by optical interactions of light with nanometer-sized structures (structural color)^3^. Melanin granules, which are produced by several enzymatic reactions of 3,4-dihydroxyphenylalanine (DOPA), are known to be one of the primary components of nanostructural elements producing structural coloration^4^. Melanin granules act both as components of structural colors and as light-scattering absorbers, producing bright structural colors^5^. Previous studies have demonstrated that the various sizes of nanostructural elements cause variations in the reflected structural colors^6^. Additionally, the arrangement of melanin granules is a key factor in controlling the two types of structural colors: iridescent color and non-iridescent color. In layers around the edges of iridescent barbules, melanin granules become close-packed structures. For example, male peacock feather colors are created by the close-packed arrays of rod-like melanin granules^4^,^7^. Turkey feather also have iridescent structural colors due to the assembly of hollow melanin granules^8^. On the other hand, the arrangements of granule like structure with some materials (e.g. keratin, pigment) show the amorphous structures in non-iridescent barb or barbules^9^. *Cotinga Maynana* bird feathers are well-known example of non-iridescent structural colors^10^.

To create artificial structural color materials, the use of lithography techniques, liquid crystals, block copolymers, and colloidal particles have all been investigated^11^. Among these, structural color materials based on the assembly of colloidal particles have been of particular interest because these techniques are easily performed and can be scaled up^12^,^13^,^14^. In previous reports, silica particles or polymer particles, such as polystyrene and poly(methyl methacrylate) particles, were usually used as a component of structural color materials. However, the structural colors produced by these materials exhibit problems with their faint colors because light scattering produces milky-white colors. To overcome this drawback, several researchers investigated the doping of black materials. Because carbon black^15^,^16^,^17^, cuttlefish ink^18^, and polypyrrole^19^ absorb the white colors produced by light scattering, bright structural colors can be obtained by increasing the feed ratio of black materials. While these methods are useful for the preparation of bright structural colors, the colors obtained were usually only non-iridescent colors because black material doping restricts the close-packed assembly of the colloidal particles. The melanin-like biomimetic polymer ‘polydopamine (PDA)’ is easily prepared from dopamine (DA), which is an amino-acid derivative, under basic conditions^20^. Very recently, we demonstrated a facile method for the fabrication of submicrometer-sized monodisperse PDA black particles that mimic melanin granules and used them as components of structural color materials^21^. Relatively monodisperse PDA black particles act both as components of structural color materials and as scattering absorbers, producing bright and non-iridescent structural colors. However, the structural colors produced by PDA black particles show dark coloration because the black particles absorb too much of the scattered light. Thus, to produce high-visibility structural colors, it remains a challenge to develop an effective strategy for the preparation of colloidal particles with any blackness. Additionally, high-visibility colors can be produced by controlling the refractive index of the nano-elements^22^. Recent studies suggest that some melanin granules are core-shell-like structures composed of materials with different refractive indices. Human melanin granules are composed of a pheomelanin core and a eumelanin outer shell^23^. Turkey melanin granules also have similar core-shell-like structures composed of air and eumelanin (hollow structures)^8^. These core-shell structures are composed of core materials with low refractive indices and shell layers with high refractive indices. Iridescent optical effects will occur at the interface of materials with different refractive indices and become more pronounced with greater differences in refractive indices^8^.

Herein, we demonstrated the facile synthesis of biomimetic core-shell particles with melanin-like shell layers (Fig. 1). Monodisperse polystyrene (PSt) particles (refractive index = 1.59^24^) were used as the core material, and PDA layers (refractive index of melanin ~1.7–1.8^25^) was selected as the shell layer. The synthesized products are designated PSt(X)@PDA(Y) core-shell particles (X: diameter of PSt core particles, Y: thickness of PDA shell layer). Although many researchers have reported the synthesis of PDA-coated materials^26^,^27^,^28^, their potential use as structural color materials have not yet been achieved. PSt@PDA core-shell particles with different blackness levels were produced by controlling and varying the thicknesses of the PDA shell layers. The pellets prepared by core-shell particles demonstrated high-visibility structural colors, and the reflection wavelength of the structural colors increased as the PDA shell thicknesses increased. The visibility of the structural colors from the PSt@PDA core-shell particles dramatically increased to the naked eye compared with that of bare PSt core particles. Both the iridescent and non-iridescent structural colors were easily manufactured by controlling and varying the PDA shell thicknesses, which caused surface roughness. In comparison with other works including the black material doping, the present method enables the creation of structural colors with high-visibility using a single component. To investigate the usability of PSt@PDA core-shell particles compared with pure PDA particles, the effects of the particle components on structural coloration were also investigated.

## Results and Discussion

### Synthesis of PSt@PDA core-shell particles

**Figure 2:**
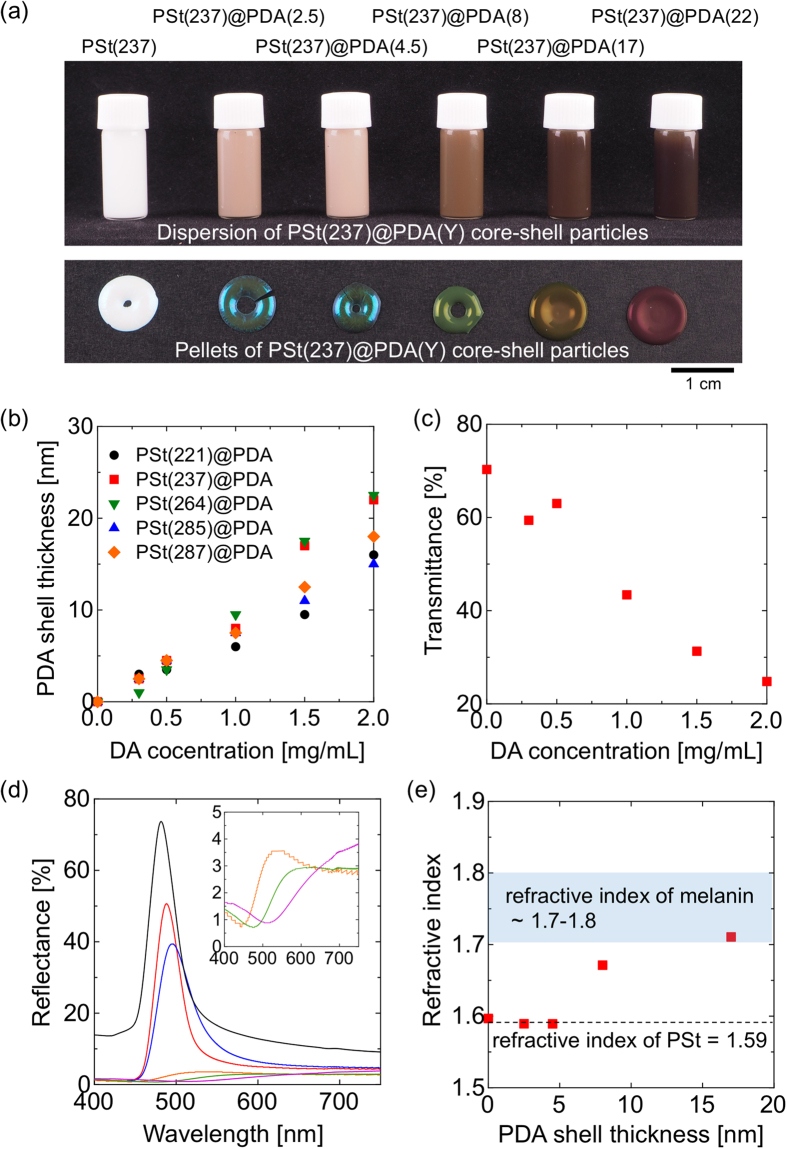
(**a**) Photographs of (upper row) dispersions of PSt(237)@PDA(Y) core-shell particles (0.5 wt% in water) and (lower row) structural color pellets made of PSt(237)@PDA(Y) core-shell particles. (**b**) The effects of DA concentration on PDA shell thickness. (**c**) Transmittances of dispersions of PSt(237)@PDA(Y) core-shell particles (0.005 wt% in water). Transmittance was recorded at *λ* = 600 nm. (**d**) Reflection spectra of structural color pellets from PSt(237)@PDA(Y) core-shell particles. Black: PSt, red: PSt(237)@PDA(2.5), blue: PSt(237)@PDA(4.5), orange: PSt(237)@PDA(8), green: PSt(237)@PDA(17), and pink: PSt(237)@PDA(22). (**e**) Refractive indices of PSt(237)@PDA(Y) core-shell particles. The refractive index (*n*) was calculated using Equation (1). *m* = 1, *θ* = 90, *λ* is the *λ*_max_ of the reflection peaks shown in Fig. 2d, and *d* is the diameter of PSt(237)@PDA(Y) particles.

Monodisperse PSt particles with different diameters, which were prepared by emulsifier-free emulsion polymerization using hydrophilic comonomers, were used as the core material^29^. According to the method in our previous report, PSt@PDA core-shell particles were prepared in the DA polymerizations performed at different monomer concentrations in the presence of PSt core particles^30^,^31^. The coating of the PDA shell layer onto the PSt core particles was confirmed using FT-IR spectroscopy (see Figure S1). While the surface ζ potential of the PSt core particles was found to be approximately +60 mV, that of the PSt@PDA(Y) core-shell particles was measured to be negative (approximately −60 mV), which also indicated the presence of PDA shell layers (see Figure S2). Due to the high ζ potentials, the dispersions obtained were very stable in water, and no significant change was observed after 3 months of storage. The upper row of Fig. 2a shows photographs of the dispersion of PSt(237)@PDA(Y) core-shell particles (0.5 wt% in water). The brown coloration increased as the DA monomer concentration increased from 0.3 to 2.0 mg/mL. The PDA shell thickness, which was measured by SEM, gradually increased from approximately 2.5 nm to approximately 22 nm as the DA concentration increased, without affecting the core size (Fig. 2b). The blackness of the particles is an important factor related to obtaining high-visibility structural colors. Thus, we measured the UV-vis spectra of 0.005 wt% water dispersions of PSt(237)@PDA(Y) core-shell particles (light path = 10 mm) to investigate their behaviors. As shown in Fig. 2c, the transmittance at 600 nm decreased as the shell thickness increased, indicating that the variation of the particles’ blackness was effectively controlled by the PDA coating method.

### Fabrication of high-visibility structural color pellets from core-shell particles

The lower row of Fig. 2a shows the structural color pellets prepared by PSt(237)@PDA(Y) core-shell particles together with the pellets from bare PSt particles. When conventional PSt particles were used as components, color pellets that were milky-white due to light scattering were obtained. In contrast, PSt@PDA core-shell particles produced bright structural color pellets. These pellets were created using only PSt@PDA core-shell particles as components. When the pellets were deliberately broken, brown powders were obtained (see Figure S3). A PDA shell layer of only 2.5 nm dramatically increased the visibility of structural colors to the naked eye. As the thickness of the PDA shell layer increased, the colors of the pellets changed from blue to green to red. Figure 2d shows the reflection spectra of the pellets. The reflection peaks underwent a red shift as the PDA layer thickness increased, in accordance with a previously reported result that structural colors were controlled by altering the size of particles^16^,^21^. A change in the PDA layer thickness also influenced the reflectance percentage. When PSt@PDA core-shell particles with thin shell layers (0–4.5 nm) were used as components, the highest reflectance of the pellets was approximately 40–80%, while the highest reflectance of the pellets made from particles with a thick PDA layer (8–22 nm) dramatically decreased to approximately 5%, at which point saturated structural colors were observed by the naked eye; this result is in good agreement with that reported previously^16^,^21^.

Under practical conditions, Bragg’s law can be approximately expressed as Equation (1) by considering the effective refractive index of the system:


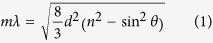


where *m* is the order of diffraction, *λ* is the wavelength of light, *n* is the refractive index of colloidal particles, *d* is the center-to-center distances between the nearest particles, and *θ* is the angle between the incident light and the diffraction crystal planes^32^. The refractive index of PSt(237)@PDA(Y) particles can be calculated using Equation (1) assuming that Bragg’s law holds. As shown in Fig. 2e, the refractive index of PSt@PDA particles with thin shell layers (0–4.5 nm) was approximately 1.59, which is relatively the same as the refractive index of PSt^24^. The refractive index of the PSt@PDA particles will be depending on the volume averaged value of PSt and PDA. When PDA shell thickness were thin, the samples would be almost similar to PSt. In contrast, the refractive index of PSt@PDA particles increased to ca. 1.7 as the PDA shell thickness increased. The refractive index value of the barbules’ melanin, measured by the Jamin-Lebedeff interference microscopy method, was approximately 1.7-1.8^25^. While matching the refractive index of PSt@PDA with that of melanin has no physical meaning, that of the PSt@PDA particles with a thick PDA layer (>17 nm) was relatively the same as that of native melanin. These data clearly indicate the usefulness of the PDA coating method for controlling the particles’ refractive index as well as their blackness.

**Figure 3:**
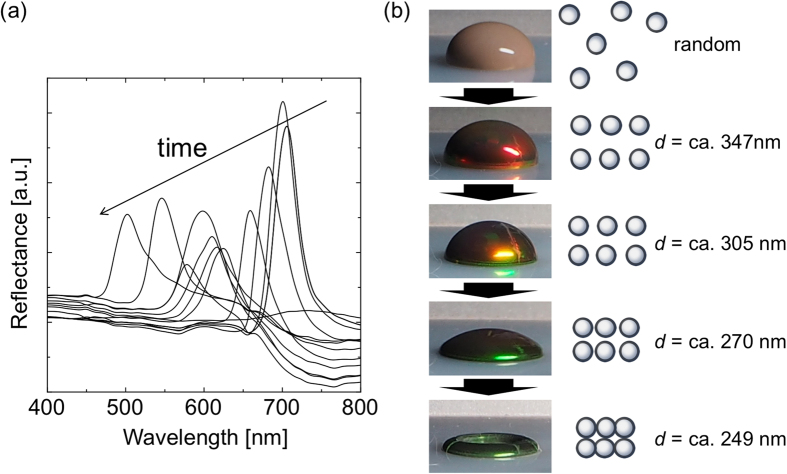
Pellet creation process from a dispersion of PSt(237)@PDA(4.5) core-shell particles. (**a**) Reflection spectrum change over time. (**b**) Time-series photographs of dispersions. The center-to-center distance between the nearest particles (*d*) was calculated using Equation (1). *m* = 1, *θ* = 90, refractive index (*n*) = 1.59, and *λ* is the *λ*_max_ of the reflection peaks shown in Fig. 3a.

The process of creating these pellets was investigated. A total of 100 μL of a water dispersion of PSt(237)@PDA(4.5) particles was dropped onto a silicone rubber plate using a micropipette, and the suspensions were allowed to dry. Structural color pellets were generally obtained at room temperature for 12 h. However, it takes a lot of time to measure the reflection spectra. Thus, in the present experiments, pellets were prepared at 50 °C for about half an hour. The reflection spectra of these dispersions were measured with a microscopic spectrophotometer as a function of the evaporation time. As shown in Fig. 3a, the *λ*_max_ of the reflection peaks decreased as time increased, clearly indicating the blue-shift of the reflection spectra. Figure 3b shows a photograph of the dispersions. Before the water evaporated, dispersions of PSt(237)@PDA(4.5) particles (5 wt% in water) were light brown in color due to absorption. During the evaporation of the water, the colors changed, and finally, a blue-green structural color pellet was obtained. The center-to-center distances between the nearest particles (*d*), calculated using Equation (1) (n = 1.59), gradually decreased from approximately 347 nm as the evaporation time increased, because of the increase of particle concentrations. Finally, *d* reached a plateau at approximately 249 nm, which is consistent with the diameter of PSt(237)@PDA(4.5) particles (246 nm), indicating the formation of close-packed structures.

**Figure 4:**
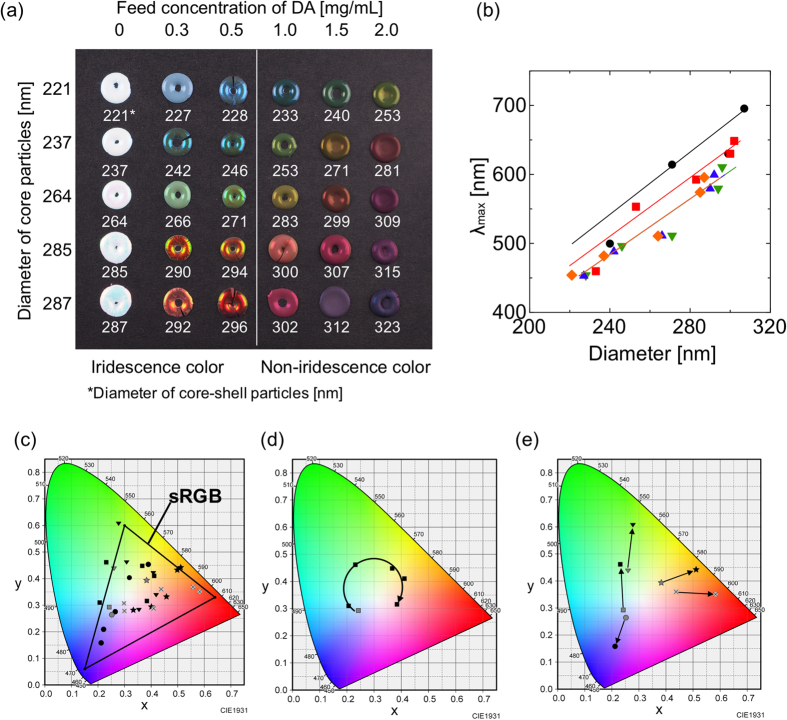
(**a**) Structural color pellets from PSt(X)@PDA(Y) core-shell particles. (**b**) *λ*_max_ of the reflection peaks of structural color pellets as a function of particle diameter. Feed concentration of DA (mg/mL): solid diamond, 0; solid triangle, 0.3; solid inverted triangle, 0.5; solid square, 1.0; and solid circle, 1.5. Theoretical lines were drawn according to Equation (1): orange, blue, and green lines, refractive index (*n*) = 1.59; red line, *n* = 1.64; and black line, *n* = 1.70. (**c**) The International Commission on Illumination (CIE) 1931 chromaticity diagram labeled with the limits of the sRGB color gamut. (**d**) The CIE chromaticity chart of pellets prepared by PSt(237) core particles with different PDA shell thicknesses. (**e**) The CIE chromaticity plots of colors prepared by PSt core particles (gray plots) and PSt(X)@PDA(Y) core-shell particles (black plots, DA concentration; 0.5 mg/mL). Circle, PSt(221); square, PSt(237); inverted triangle, PSt(264); star, PSt(285); and x, PSt(287).

By selecting the core size and feed concentration of DA monomers, we obtained pellets in a variety of colors (Fig. 4a). The reflectance spectra of each of the structural color pellets were measured by the microscopic spectrophotometer. The *λ*_max_ values of the reflection peaks of the pellets were plotted as a function of the diameter of the core-shell particles (Fig. 4b). The *λ*_max_ of the reflection peaks increased as the diameters of the core-shell particles increased. As the PDA shell thicknesses are controlled by the feed concentration of the DA monomer (Fig. 2b), the refractive indices of particles prepared under the same monomer conditions will be relatively similar. Thus, the theoretical lines in Fig. 4b were calculated from Equation (1) using the refractive indices for PSt(237)@PDA(Y) particles (Fig. 2e). The model values correspond reasonably well with the experimental data, suggesting that structural coloration was controlled by the refractive indices and the diameters of the particles.

In Fig. 4c, the colors of each pellet are plotted on the International Commission on Illumination (CIE) 1931 chromaticity diagram labeled with the limits of the sRGB color gamut. By controlling the diameters of the core particles and PDA shell thicknesses, we were able to obtain nearly the full range of colors. Figure 4d shows the CIE chromaticity chart for the pellets prepared by PSt(237) core particles with different PDA shell thicknesses. The colors obtained indicated a gradual transition from the blue to the green to the red portions of the visible spectrum. Next, we investigated the effect of the PDA coating on the colorfulness of the structural colors. Figure 4e shows plots of the colors prepared by PSt core particles (gray plots) and PSt(X)@PDA(Y) core-shell particles (black plots, DA concentration = 0.5 mg/mL). After PDA coating, the colors moved to the outside of the plot, indicating the increase of colorfulness. These results strongly indicate the ability of the present method to create high-visibility structural color materials with a full color range.

**Figure 5:**
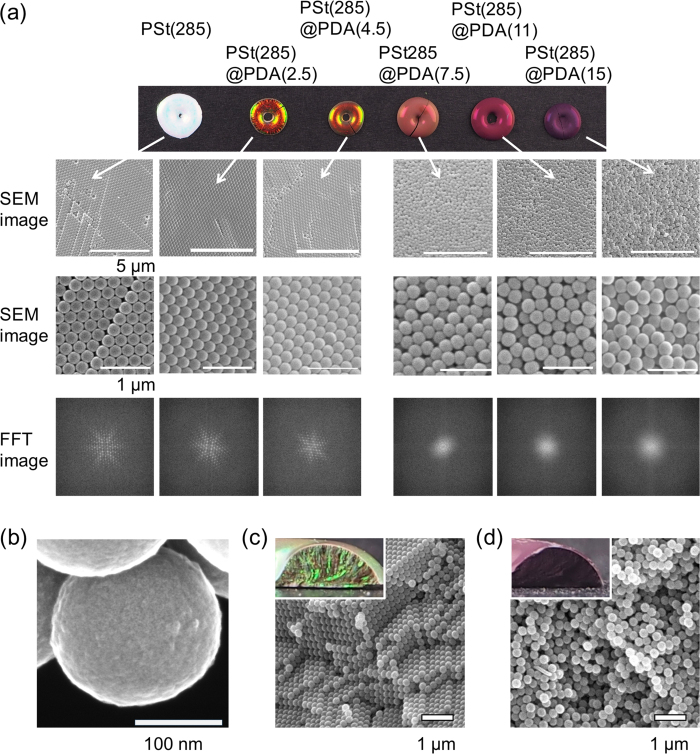
(**a**) SEM images and FFT spectra from SEM images of structural color pellets prepared by PSt(285)@PDA(Y) core-shell particles. FFT images were obtained from lower SEM images. (**b**) High resolution FE-SEM image of PSt(285)@PDA(15) particles. (**c**) Cross section view of PSt(285)@PDA(2.5) pellets. Inset shows a photograph of the samples. (**d**) Cross-section view of PSt(285)@PDA(15) pellets. Inset shows a photograph of the samples.

**Figure 6:**
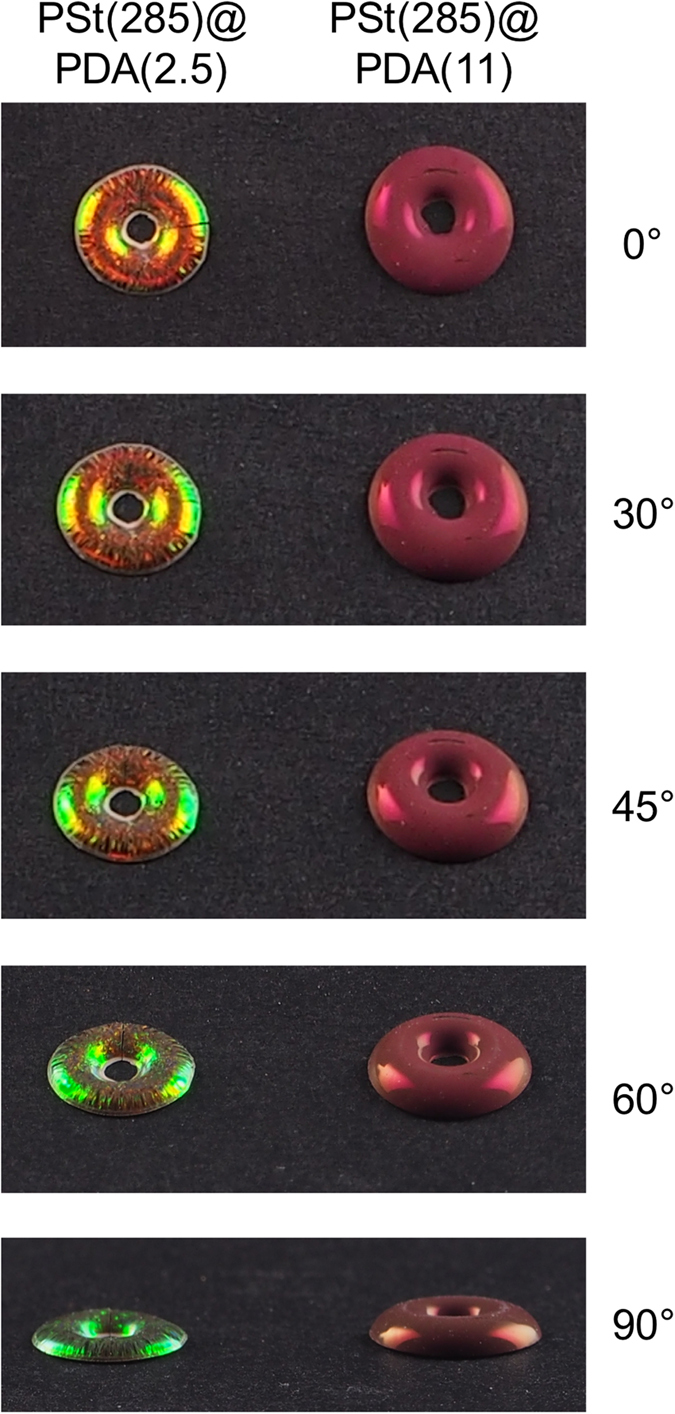
Photographs of structural color pellets from PSt(285)@PDA(2.5) and PSt(285)@PDA(11) core-shell particles viewed from different viewing angles.

Figure 4a shows that two types of colors were obtained: iridescent and non-iridescent structural colors. When the PDA shell thickness was thin, the obtained pellets exhibited iridescent structural colors (Fig. 5). In contrast, the core-shell particles with thick shell layers produced non-iridescent structural color pellets (Fig. 5). This result is likely due to the arrangement of the particles. We performed SEM measurements of the pellet surface to investigate the arrangement of the particles (Fig. 6a). Iridescent structural colors were obtained from colloidal crystal structures, and non-iridescent colors were obtained from amorphous structures. Fourier transforms (FFT) of the SEM images of the pellets were investigated^14^,^16^,^21^. Sharp hexagonal peaks were observed for colloidal crystals, indicating the formation of close-packed structures. In contrast, circular patterns were produced by roughly packed arrays, forming amorphous structures. In the amorphous state, diffraction will be suppressed and the reflection spectra related to the size of the particles will be selectively enhanced, producing non-iridescent structural colors^14^. These results clearly indicated that two type of colors were observed for the different particle arrangements. To investigate the effects of thick PDA shell layers on the arrangement of particles, high resolution field emission-SEM (FE-SEM) images of PSt(285)@PDA(15) particles were taken (Fig. 6b). While the coefficient of variation (CV) of the particles remained approximately 3%, the PSt(285)@PDA(15) core-shell particles had rough surfaces. DA polymerization occurs in both the surface of the particles and in the solution phase. When the monomer concentration was high, DA polymerization primarily occurred in the water phase. The resulting DA oligomers would aggregate onto the particles, producing a rough surface. The surface roughness of these particles would prevent the production of colloidal crystal structures. Figure 6c,d shows the cross-section of the pellets. While close-packed structures were observed in PSt(285)@PDA(2.5) pellets (Fig. 6c), amorphous structures were obtained in PSt(285)@PDA(15) pellets (Fig. 6d), indicating the uniformity of the pellets. These results indicated that both iridescent and non-iridescent high-visibility structural colors were easily prepared using the same process. Therefore, this method enables the separate formation of colloidal crystal structures and amorphous colloidal structures by core-shell particles whose only difference is their shell thicknesses. The simplicity of our method facilitates the practical use of these particles because this method did not require any additives.

**Figure 7:**
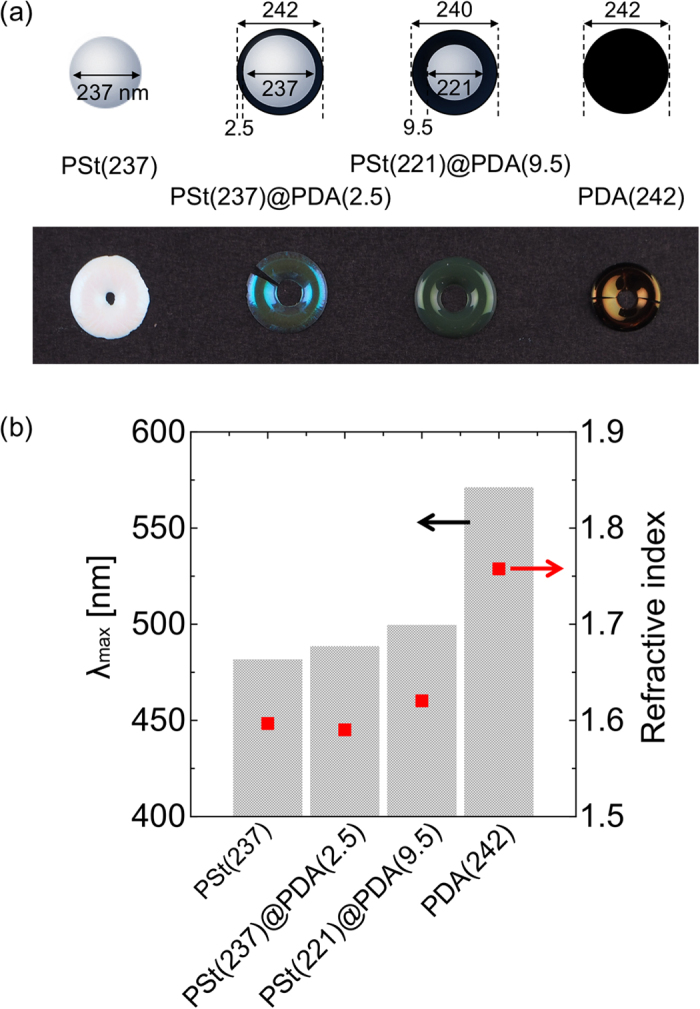
(**a**) Photographs of structural color pellets produced from PSt(237), PSt(237)@PDA(2.5), PSt(221)@PDA(9.5), and PDA(242) particles. (**b**) *λ*_max_ of the reflection peaks and refractive indices of the particles shown in Fig. 6a.

Finally, we examined the effects of particle components on structural coloration. To investigate the effects of the ratio of the PSt region and the PDA region, four samples were prepared: PSt(237), PSt(237)@PDA(2.5), PSt(221)@PDA(9.5), and PDA(242) particles. While the four particles have relatively similar diameters, the colors of the pellets were different (Fig. 7a). As shown in Fig. 7b, the *λ*_max_ of the reflection peaks, as measured by the microscopic spectrophotometer, increased as the PDA regions increased. The variation in the *λ*_max_ of the reflection peaks is due to variations in the refractive index of the particles. Figure 7b also shows the refractive index of the particles as calculated using Equation (1). The refractive index of the particles gradually increased as the PDA ratio increased. While it is difficult to accurately determine the refractive index for PDA, which is a strongly adsorbing polymer, the refractive index of PDA particles was calculated to be approximately 1.76, which is in agreement with a previous report^33^. For the refractive index, a similar trend is observed with *λ*_max_, strongly indicating that the structural coloration produced by colloidal particles depends on their refractive index.

In conclusion, we developed both iridescent and non-iridescent high-visibility structural colors from biomimetic colloidal particles. This process was inspired by the structural color found in bird feathers. To create high-visibility structural colors, we designed and synthesized PSt@PDA core-shell particles that met the following four conditions: size, blackness, refractive index, and arrangement. By controlling the feed concentration of the DA monomers, core-shell particles with any size, blackness, and refractive index were easily prepared under mild conditions. Additionally, the surface roughness of the particles, which is an important factor for controlling the arrangement of the particles, was adjusted by varying the PDA thickness. While PSt@PDA particles with smooth surfaces produced colloidal crystal structures, PSt@PDA particles with rough surfaces produced amorphous structures. As a result, both iridescent and non-iridescent structural colors were separately prepared from only one component. Because PSt@PDA core-shell particles have properties similar to those of melanin, this method will be useful for understanding biological systems in nature. Furthermore, this method enables the production of high-visibility structural colors with a simple process. Additional studies in progress include the development of structural color inks based on this methodology. The results of these investigations will be described in future reports.

## Methods and Material

### Materials

Dopamine hydrochloride (DA) was obtained from Sigma-Aldrich. Tris(hydroxymethyl)aminomethane (Tris) was obtained from Kanto Chemical. Deionized water with a resistance of 18.2 MΩ was obtained using a Millipore Simplicity UV system. Highly monodisperse PSt core particles with different diameters^29^ and PDA black particles^21^ were prepared according to the respective methods reported in the literature.

### Measurements

Infrared (IR) spectra were measured by Fourier transform infrared (FT-IR) spectroscopy (FTIR-420, JASCO). Ultraviolet-visible (UV-vis) spectra were obtained using a spectrophotometer (U-3010; Hitachi). The hydrodynamic diameter (*D*_h_) and ζ potentials of particles were measured by dynamic light scattering (DLS) (ELSZ; Otsuka Electronics). Scanning electron microscopy (SEM) micrographs of the samples were obtained using a scanning electron microscope (JSM-6510A; JEOL). Reflection spectroscopies were performed using a spectrophotometer (V-650; JASCO) equipped with a reflection spectroscopy unit (ARSV-732; JASCO) and a microscopic spectrophotometer (MSV-370; JASCO). Photographs of the samples were taken with a digital camera (OM-D, Olympus).

### Synthesis of PSt@PDA core-shell particles

DA (36–240 mg, 0.19–1.27 mmol), Tris (1.45 g, 12 mmol), and PSt particles (0.12 g) dispersed in deionized water (20 mL) were stirred for 20 h at room temperature. The core-shell particles were separated and purified by repeated centrifugation (14,500 rpm for 20 min) and redispersion.

### Preparation of structural color pellets from PSt@PDA core-shell particles

Structural color pellets were fabricated by pouring 10 wt% PSt@PDA core-shell particle suspensions onto a silicone rubber plate and allowing the suspensions to dry at room temperature for 12 h.

## Additional Information

**How to cite this article**: Kawamura, A. *et al.* Full-Color Biomimetic Photonic Materials with Iridescent and Non-Iridescent Structural Colors. *Sci. Rep.* **6**, 33984; doi: 10.1038/srep33984 (2016).

## Supplementary Material

Supplementary Information

## Figures and Tables

**Figure 1:**
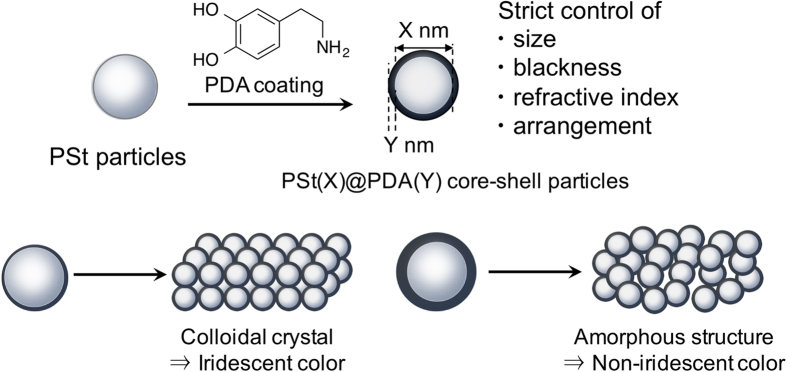
Schematic representation of the fabrication of core-shell particles with biomimetic melanin-like PDA shell layers.
