# Supplementary material for: Full-Color Biomimetic Photonic Materials with Iridescent and Non-Iridescent Structural Colors
Source: Sci Rep. 2016 Sep 23;6:33984. doi: 10.1038/srep33984 (PMC5034286; doi:10.1038/srep33984)
Supplement: Supplementary Information [file srep33984-s1.pdf]

Supporting Information

Full-Color Biomimetic Photonic Materials with Iridescent and Non-Iridescent Structural Colors

Ayaka Kawamura, Michinari Kohri, Gen Morimoto, Yuri Nannichi, Tatsuo Taniguchi, and Keiki Kishikawa*

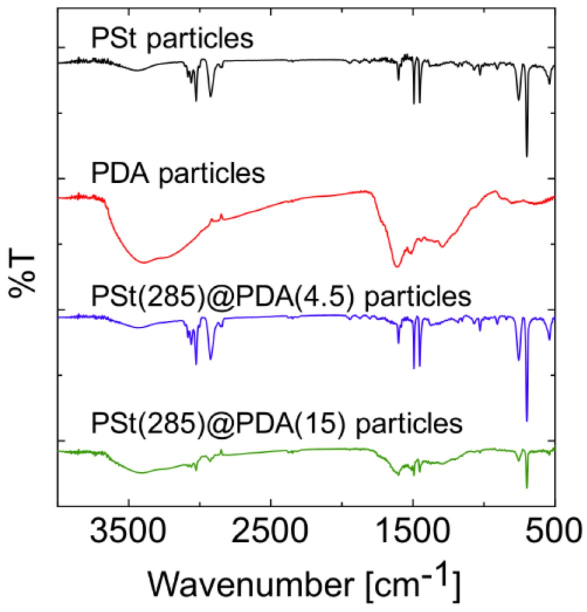

Figure S1 IR spectra of PSt, PDA, PSt(285)@PDA(4.5), and PST(285)@PDA(15) particles.

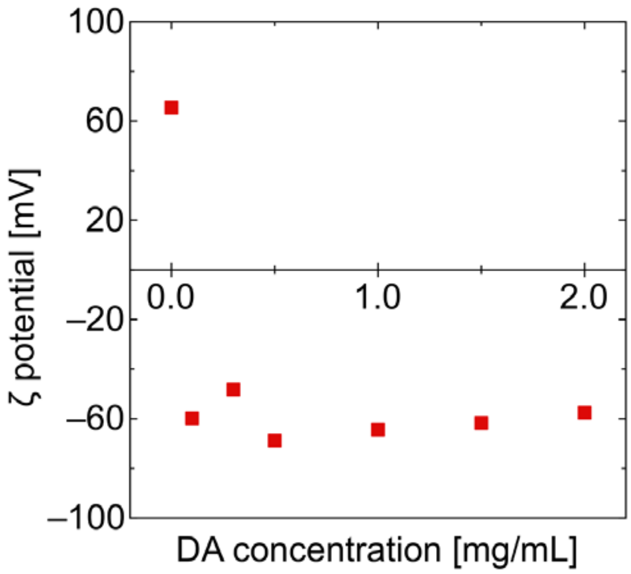

Figure S2 ζ potentials of particles as a function of feed concentration of DA monomer.

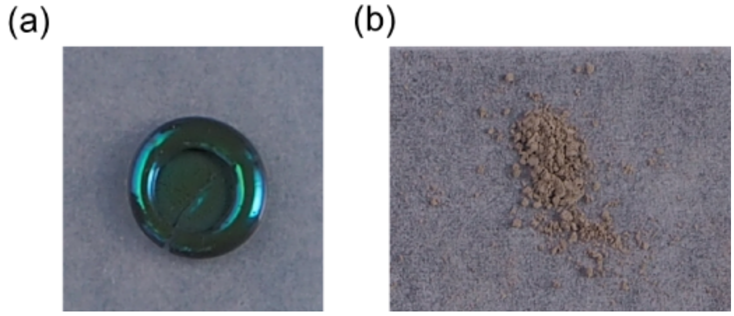

Figure S3 Photographs of (a) structural color pellets from PSt(237)@PDA(4.5) core-shell particles and (b) deliberately broken samples.
